# Proposal to refine the mechanistic classification of chronic pain and corresponding clinical symptomatology

**DOI:** 10.3389/fpain.2026.1963056

**Published:** 2026-09-21

**Authors:** Jean-Pascal Lefaucheur

**Affiliations:** 1 UR4391 (ENT Team), Faculty of Health, Paris Est Créteil University, Créteil, France; 2 Department of Clinical Neurophysiology, Henri Mondor University Hospital, AP-HP, Créteil, France

**Keywords:** chronic pain, diagnosis, large fibers, neuropathic, nociceptive, nociplastic, sensitization, small fibers

## Abstract

The management and treatment of patients suffering from chronic pain must be based on a precise understanding of the pathophysiological mechanisms underlying the syndrome. However, it cannot be defined solely by the presence or absence of disease or injury affecting either non-neuronal tissues or the neuronal structures of the somatosensory system, as these elements do not always accurately or causally reflect the underlying mechanisms of the pain experienced by patients. The current taxonomy of chronic pain classically includes three main mechanisms: nociceptive, neuropathic, and nociplastic. However, certain ambiguities of this classification need clarification, such as: (1) the importance given to demonstrating a lesion of the somatosensory nervous system for the diagnosis of neuropathic pain; (2) the specificity of sensory descriptors for the diagnosis of neuropathic pain; (3) the imprecision of the concept of “central sensitization”. In this article, a new taxonomy is proposed, identifying: (1) differential mechanisms of neuropathic pain related to “large” or “small” nerve fiber systems; (2) “sensory”, “cognitive”, and “emotional” components of nociplastic pain; (3) “sensory nociplastic pain”, which can be distinguished from neuropathic pain by the presence of diffuse, variable, and multiform “neuropathic-like” symptoms; (4) a modulator linked to sensitization related to psychosocial vulnerability and stressors. A comprehensive understanding of the mechanisms of pain relies on a rigorous interpretation of patients’ symptoms. It is particularly important to determine whether sensitization mechanisms are present from disease onset, suggesting primary nociplastic pain, or whether they emerge over time, reflecting secondary processes of nociceptive or neuropathic pain syndrome chronification.

## Introduction

In 2017, the International Association for the Study of Pain (IASP) proposed a classification of chronic pain based on three main mechanisms, which has since been unanimously adopted in clinical practice. These three mechanisms are called nociceptive, neuropathic, and nociplastic (1). Nociceptive pain is a “pain that arises from actual or threatened damage to non-neural tissue and is due to the activation of nociceptors” occurring with a normally functioning somatosensory nervous system (1). In a way, nociceptive pain, such as that associated with chronic inflammatory joint diseases, continues to play an alarm and protective role for the affected body part, like a kind of “prolonged acute pain”. Neuropathic pain is a “pain caused by a lesion or disease of the somatosensory nervous system” (1). The injury or disease may be located in the peripheral nervous system (e.g., nerve trunk or plexus injury, or polyneuropathy) or in the central nervous system (e.g., spinal cord injury or stroke). Various clinical questionnaires have been proposed for the diagnosis of neuropathic pain, as well as the use of complementary tests (2), but none of these approaches is really satisfactory and specific for this diagnosis (3, 4). Nociplastic pain is a “pain that arises from altered nociception despite no clear evidence of actual or threatened tissue damage causing the activation of peripheral nociceptors or evidence for disease or lesion of the somatosensory system causing the pain” (1). Nociplastic pain is classically characterized by the existence of central sensitization processes (5), but the term “central sensitization” is very imprecise and covers a wide variety of pathophysiological mechanisms (6). In addition, central sensitization can lead to patients experiencing a variety of sensory symptoms, including “neuropathic-like” symptoms, making it difficult to differentiate between nociplastic and neuropathic pain (3). Thus, this classification based on this triple model appears imperfect, and in particular insufficiently precise given the plurality of mechanisms involved in chronic pain and the associated clinical symptoms. Therefore, a new taxonomy of chronic pain and its corresponding clinical phenotypes is proposed in this text.

## Nociceptive pain and hyperalgesia

The concept of nociceptive pain is certainly the easiest to grasp, as it relies directly on the principles of acute pain, which is a response to harmful stimuli, associated with tissue damage that poses a threat to the body. Like acute pain, nociceptive pain is primarily expressed in terms of intensity, or by descriptors such as sharp, deep, bruising, squeezing, cramping, pressing, aching, throwing, stabbing, stretching, pulling, throbbing, pounding, tearing, grinding, gnawing, biting, pinching, twinging, thrusting, soreness, or localized pressure pain.

The diagnostic definition of the mechanisms of chronic pain relies primarily on the evaluation of two clinical components: the descriptors of spontaneous pain and the characterization of “evoked pain” phenomena, if they exist. In the context of nociceptive pain, the persistent activation of nociceptors, induced by inflammatory mediators for example, can trigger a sensitization mechanism at the peripheral level, which can be the cause of evoked pain. This sensitization process is linked to a variety of posttranslational mechanisms (7), such as changes in membrane expression or trafficking of nociceptors, or changes in the activation thresholds and kinetics of associated ion channels. This can lead to increased axonal excitability or impaired intra-axonal signal transduction and, ultimately, central changes in the integration of afferent nociceptive information in the spinal cord (8). Clinically, in addition to spontaneous ongoing pain, this manifests as hyperalgesia, which is an intensification of pain elicited by a normally painful stimulus. Hyperalgesia can therefore constitute an important symptomatic dimension of nociceptive pain.

## Different types of neuropathic pain

Neuropathic pain is linked to a “lesion or disease” of the somatosensory nervous system. In fact, this sensory system corresponds to a triple-system model, which comprises three independent pathways until their arrival in the somatosensory cortex. The first system consists of very large-diameter sensory fibers (A*α* fibers) originating from muscles, tendons, or joints, giving rise to the central spinocerebellar and dorsal column–medial lemniscus pathways, and responsible for “proprioception” (deep sensation). The second system consists of large-diameter sensory fibers (A*β* fibers) originating from the skin, giving rise to the dorsal column–medial lemniscus pathways, and responsible for “mechanical” cutaneous sensations (fine touch, two-point tactile discrimination, vibration). The third system consists of small diameter sensory fibers (thinly myelinated A*δ* fibers and unmyelinated C fibers) originating from the skin or internal organs, giving rise to the extralemniscal spinothalamic pathways, and responsible for “thermal” or “painful” cutaneous sensations (innocuous cold or warm sensations, and nociceptive sensations) and visceral sensations.

Damage to these nerve fibers or pathways results in a sensory deficit (negative signs and symptoms). For example, damage to the very large A*α* sensory fibers leads to impaired arthrokinesis, decreased deep tendon reflexes, or proprioceptive ataxia, with instability when standing and walking and an increased risk of falls. Damage to the large A*β* sensory fibers (or dorsal column–medial lemniscus pathways) causes hypoesthesia to touch and vibration (hypopallesthesia) and sensations of numbness or walking on cotton. Damage to the small A*δ* and C sensory fibers (or spinothalamic pathways) causes hypoesthesia for pain and temperature (hypoesthesia to cold stimuli for A*δ* fibers and to warm/heat stimuli for C fibers).

Conversely, pain is a positive symptom, which cannot be attributed to a loss of function, but rather to a gain of function (hyperactivity) concerning sensory afferents or their corresponding central pathways. In the triple-component model of the somatosensory nervous system, a gain of function in very large fiber system cannot be implicated in a painful phenomenon. However, chronic pain can involve a gain of function in either large or small fiber systems. This distinguishes “pathological” chronic pain from “physiological” acute pain, which involves only small fibers.

In addition, what is extremely important to consider is that the sensory descriptors of pain differ depending on whether large or small fiber system is involved. Thus, in terms of positive signs and symptoms, the involvement of large A*β* fibers is responsible for paresthesia described as electric shocks (9), which may or may not be perceived as painful, and for constricting pain, as well as allodynia to mechanical stimuli (touch, pressure). The involvement of small A*δ* and C fibers is the cause of painful sensations such as prickling, tickling, stinging, pins and needles, walking on stones, and in particular burning sensations or even painful cold or itching for the C fibers, as well as allodynia to thermal stimuli or light skin brushing, or hyperalgesia (10).

It is also important to consider that these two somatosensory systems are not sensitive to the same types of injury. The large fiber system is particularly sensitive to compression (11), whether affecting the large fibers in the nerve trunks or the dorsal columns related to spinal canal stenosis. The small fiber system is particularly sensitive to ischemia or inflammation (12), whether affecting the small fibers in the nerve trunks or the spinothalamic tracts in the anterolateral regions of the spinal cord. Therefore, a dissociated involvement of these two somatosensory systems, with clinical descriptors specific to only one system or the other in a given patient, will be particularly suggestive of neuropathic pain, especially if the spatial distribution of the symptoms is consistent with a neuroanatomical territory.

Finally, it is important to emphasize that the finding of a lesion or disease of the somatosensory nervous system in a patient suffering from chronic pain does not automatically establish a causal link with this pain, nor does it allow for the conclusion of a neuropathic pain syndrome. Such a conclusion will be based exclusively on the interpretation of all clinical elements, and in particular on the phenotype of the pain symptoms. Actually, a lesion of the somatosensory nervous system can cause a sensory deficit that is not accompanied by pain, while certain disorders of the somatosensory nervous system can cause neuropathic pain through changes in nervous structure excitability without any structural lesions visible on complementary tests (4). This is the case with neuralgia, a condition in which the excitability of nerve fibers can be increased without being accompanied by obvious structural damage. Neuralgia can be focal, linked for example to the mechanical irritation of large nerve fibers, as in classical trigeminal neuralgia due to neurovascular compression (13), or more diffuse, of various origins, affecting small nerve fibers (10).

## Nociplastic pain and central sensitization

The characteristics of nociplastic pain extend beyond the somatosensory domain and encompass various cognitive (attentional) and affective (emotional) aspects. They correspond to a triple-network model of nociceptive information integration, based on three “pain matrices” (14): a first-order “nociceptive” or “sensory” network, a second-order “cognitive” or “attentional” network, and a third-order “affective” or “emotional” network. Thus, the clinical symptomatology of nociplastic pain can be integrated into or associated with these three domains.

In the sensory domain, the symptomatology of nociplastic pain is characterized by the diffusion and multiplicity of symptoms. These symptoms can be “neuropathic-like”, which complicates the differential diagnosis with neuropathic pain, particularly when using conventional neuropathic pain questionnaires (3). Most often, these “neuropathic-like” symptoms are characterized by the absence of a systematic neurological anatomical distribution and by a variability in their quality and location over time. Sensory nociplastic pain can also be characterized by diffuse hypersensitivity to various types of external stimuli, whether sensory (manifesting as allodynia or hyperalgesia), visual, or auditory. Thus, sensory nociplastic pain is characterized by diffuse, multifaceted, and variable sensory symptoms.

In the cognitive domain, the symptomatology of nociplastic pain includes impairment of attention, concentration, memory, learning, and communication capacities, executive functions, and sleep quality, leading to chronic fatigue and a significant impact on daily functioning. Patients complain that pain diverts their attention, prevents them from concentrating or performing tasks properly, reduces their learning or communication abilities, overloads their memory, impairs their executive functions (planning, decision-making, problem-solving, or mental flexibility), disrupts their sleep, depletes their energy, or hinders their daily functioning.

In the emotional domain, the symptomatology of nociplastic pain encompasses all aspects related to anxiety or depression. Pain will be expressed as excruciating, exasperating, worrying, frustrating, unbearable, haunting, obsessive, oppressive, demoralizing, depressing, frightening, dreadful, unlivable, or even suicidal.

Nociplastic pain stems from phenomena of “central sensitization” (5), a term encompassing various pathophysiological mechanisms, as described in detail in a previous article (6). Briefly, three main mechanisms can be distinguished: (i) a “spinal sensitization” mechanism, located in the dorsal horns of the spinal cord, and leading to evoked pain symptoms (hyperalgesia/allodynia), as described in previous sections on nociceptive and neuropathic pain, but not explaining the diffuse pain observed in nociplastic pain; (ii) a “pain-related brain network sensitization” mechanism, involving the triple-network model based on pain matrices mentioned above and including or not a dysfunction of endogenous pain control systems, such as the “descending pain modulatory system”; (iii) a “psychosocial-related sensitization” mechanism, reflecting the fact that nociplastic pain is strongly influenced by a combination of stressors (personal, social, or environmental), acting as triggering events, and a possible pre-existing psychosocial vulnerability. This vulnerability is linked to the patient's personal history, negative life events, genetic predispositions, neurodevelopmental abnormalities, certain personality traits, maladaptive beliefs, or social stigma or isolation. This leads to various states of thought that can intensify the perception of pain, such as hypervigilance, inflexibility, catastrophizing, or low self-efficacy or perceived control (15). These factors can fuel the vicious cycle linking pain, cognitive impairment, stress, anxiety, depression, and altered daily functioning in a biopsychosocial model (16).

One of the most widely used questionnaires for clinically assessing central sensitization, the Central Sensitization Inventory (CSI) (17), includes diagnostic items in the sensory domain, such as “I feel pain all over my body” or “I am sensitive to bright lights”; the cognitive domain, such as “I have difficulty concentrating” or “I have difficulty remembering things”; the emotional domain, such as “I have anxiety attacks” or “I feel sad or depressed”; and the psychosocial domain, such as “I suffered trauma as a child” or “I need help in performing my daily activities.” Thus, the central mechanisms of “pain-related brain network sensitization” and “psychosocial-related sensitization” clearly reflect the multidimensional nature of chronic nociplastic pain syndrome assessed by the CSI.

These mechanisms can be “primary” in the context of typical nociplastic pain conditions, such as fibromyalgia, which occur “despite no clear evidence of actual or threatened tissue damage causing the activation of peripheral nociceptors or evidence for disease or lesion of the somatosensory system causing the pain”, according to the IASP definition. On the other hand, these alterations can also constitute a secondary mechanism involved in the chronification of pain, with its multidimensional aspects and its impact on activities of daily living, in cases of initially nociceptive or neuropathic pain syndromes. The chronicity of pain is then accompanied by changes in clinical presentation, with more diffuse and multifaceted symptoms that vary over time and space.

## Discussion

Determining the pathophysiological mechanisms underlying chronic pain syndrome is essential for its management and constitutes a major clinical challenge (18). Here, the traditional triple-mechanism model proposed by the IASP has been refined, primarily by identifying differential neuropathic pain mechanisms linked to “large fiber system” versus “small fiber system” on the one hand; and by distinguishing the “sensory”, “cognitive”, “emotional”, and “psychosocial” components of nociplastic pain on the other hand. These distinctions are of major importance because they reflect specificities of the clinical symptomatology from which the mechanisms of chronic pain can be accurately deduced, as summarized in Figure 1.

**Figure 1:**
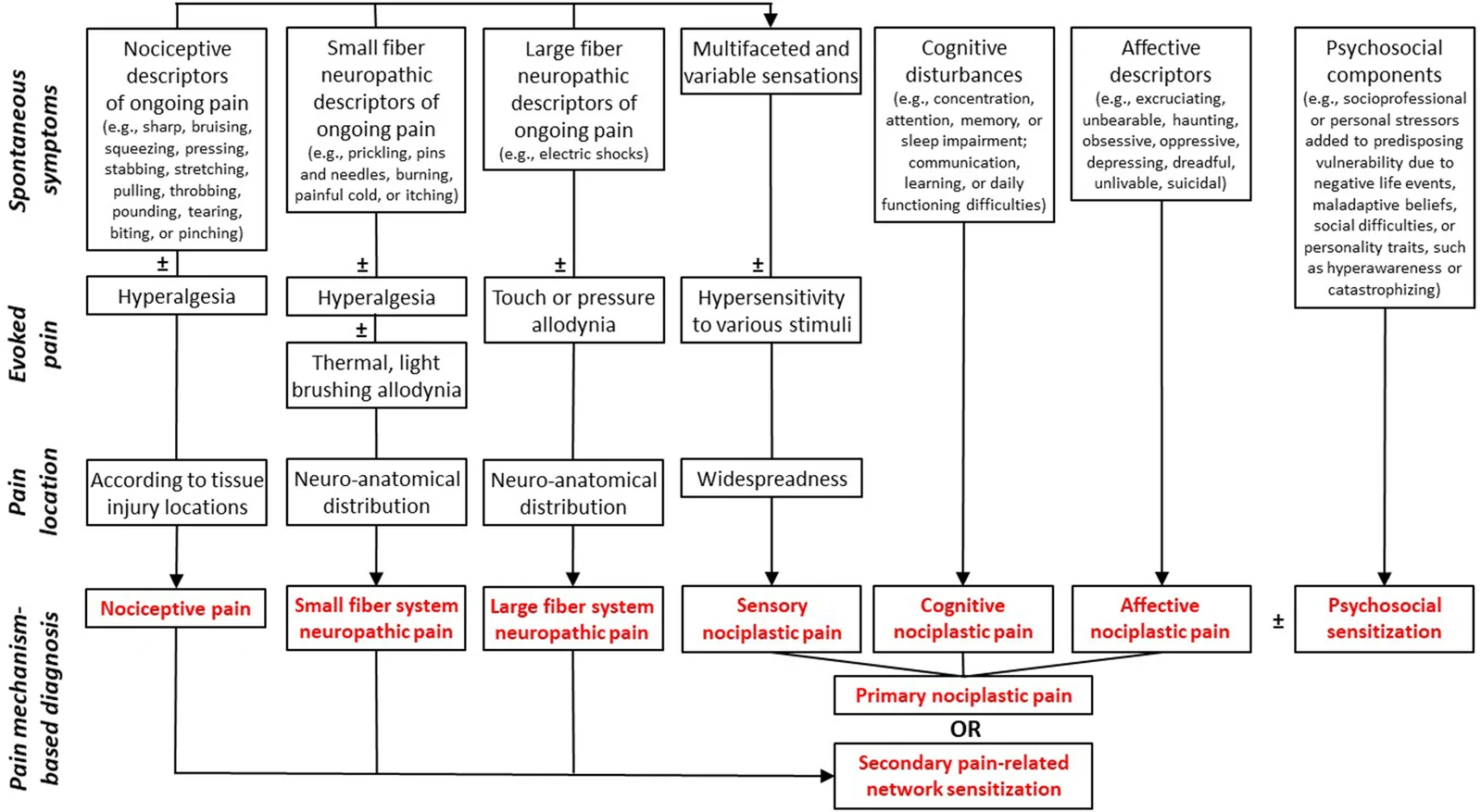
Mechanistic classification of chronic pain syndromes and corresponding clinical symptomatology.

In the sensory domain, it is crucial to distinguish “neuropathic pain” from “sensory nociplastic pain” to establish an accurate diagnosis, something that cannot be achieved using conventional neuropathic pain questionnaires (3). Neuropathic pain is suggested by the patient's history, the specificity of sensory descriptors (related to “large fiber” or “small fiber” systems), and the distribution of signs and symptoms consistent with a neuroanatomical territory (4). Sensory nociplastic pain is suggested by diffuse and multifaceted “neuropathic-like” symptoms varying over time, and nonspecific hypersensitivity to various stimuli (5). In other domains, it is also important to clearly identify and differentiate the cognitive, emotional, and psychosocial aspects of the chronic pain syndrome, as they strongly influence its therapeutic management. For example, affective symptoms can be managed with pharmacological treatments, unlike cognitive symptoms. Psychosocial components also require specific management. Conversely, it is not certain that the treatment of “primarily neuropathic” painful sensory symptoms versus “neuropathic-like” (nociplastic) painful sensory symptoms requires differentiated therapeutic approaches.

In conclusion, the mechanisms of chronic pain can be deduced from clinical symptoms, but the current taxonomy, based on a triple-mechanism model, needs refinement and certain ambiguities need clarification, particularly regarding neuropathic and nociplastic pain conditions. Specifically, the following points need to be mitigated: (i) the emphasis placed on demonstrating a lesion of the somatosensory nervous system for the diagnosis of neuropathic pain (whereas neuropathic pain symptoms may simply result from pathological abnormalities in the excitability of these nerve structures without “structural” lesion); (ii) the specificity of sensory descriptors for the diagnosis of neuropathic pain (whereas similar descriptors can be observed in the context of nociplastic pain); and (iii) the concept of central sensitization for the diagnosis of primary nociplastic pain (whereas the term “central sensitization” is used in the context of very diverse pathophysiological mechanisms of chronic pain).

Chronic pain syndrome is not limited to changes in the activity of sensory neurons and pathways. It is a multidimensional experience (sensory, cognitive, affective) influenced, to varying degrees, by psychological vulnerability and social stressors. A comprehensive understanding of this complex condition and its underlying mechanisms is based on attentive listening to the patients and a careful interpretation of the symptoms they report, ensuring that they are contextualized within the patient's medical history. It is particularly essential to distinguish between sensitization mechanisms present from the onset of the disease, which is suggestive of primary nociplastic pain, versus secondary sensitization mechanisms that appear over time, which may contribute to the chronification of nociceptive or neuropathic pain syndromes.

## Data Availability

The original contributions presented in the study are included in the article/Supplementary Material, further inquiries can be directed to the corresponding author.
